# Supplementary material for: Toxoplasma gondii infections are associated with costly boldness toward felids in a wild host
Source: Nat Commun. 2021 Jun 22;12:3842. doi: 10.1038/s41467-021-24092-x (PMC8219747; doi:10.1038/s41467-021-24092-x)
Supplement: Supplementary file 1 — Supplementary Information [file 41467_2021_24092_MOESM1_ESM.pdf]

Supplementary Material

Toxoplasma gondii infections are associated with costly boldness toward felids in a wild host

Eben Gering[†], Zachary M. Laubach[†‡*], Patty Sue D. Weber, Gisela Soboll Hussey, Kenna D. S. Lehmann, Tracy M. Montgomery, Julie W. Turner, Wei Perng, Malit O. Pioon, Kay E. Holekamp & Thomas Getty

*These authors contributed equally to this work
†Correspondence and requests for materials should be addressed to zachary.laubach@colorado.edu

‡Current address: Department of Ecology and Evolutionary Biology, University of Colorado, Boulder, USA.

This PDF file includes:

Supplementary Figures 1-4
Supplementary Tables 1-2

Other Supplementary Information for this manuscript include the following:

Supplementary Movie 1

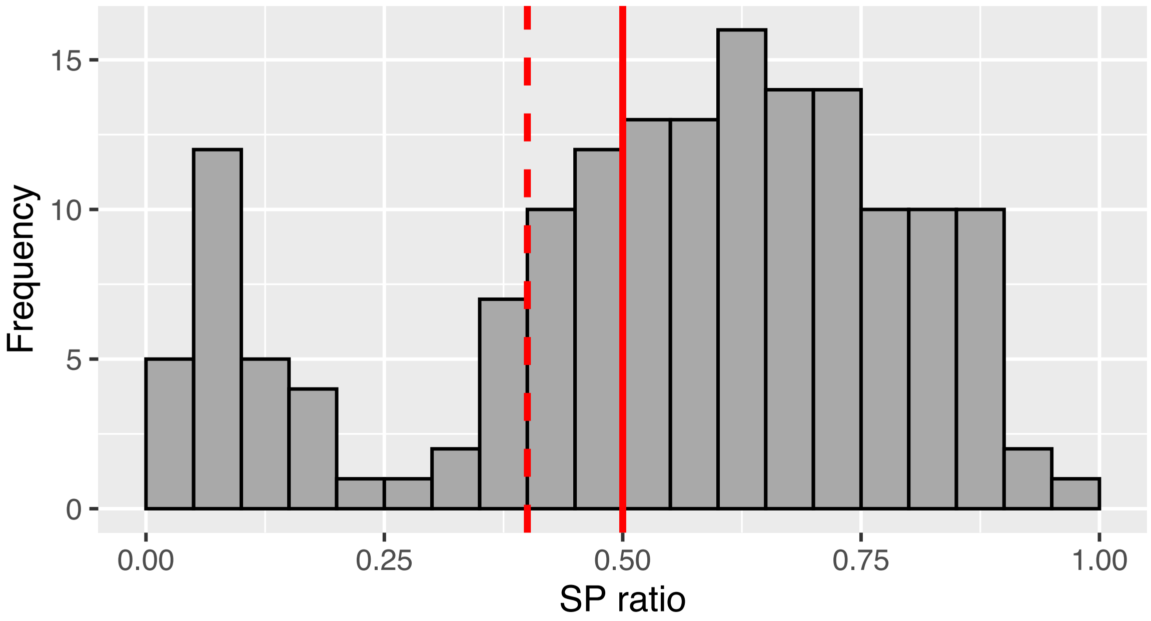

Supplementary Figure 1. Distribution *T. gondii* ELISA results (SP ratios) from spotted hyenas sampled in or near Kenya's Masai Mara National Reserve. The SP ratios are calculated as colorimetric signal of immunoreactivity for a tested blood sample (S) divided by that of a positive control (P), after subtracting the background signal for the ELISA plate (i.e. a negative control) from both S and P. The dashed red line is the upper SP ratio cutoff for negative diagnoses (SP ratio ≤ 0.40), and the solid red line is the lower cutoff for positive diagnosis (SP ratio ≥ 0.50). The region between the red lines corresponds to the SP ratio range for "doubtful," which were treated as negative in this study. Source data are provided as a Source Data file.

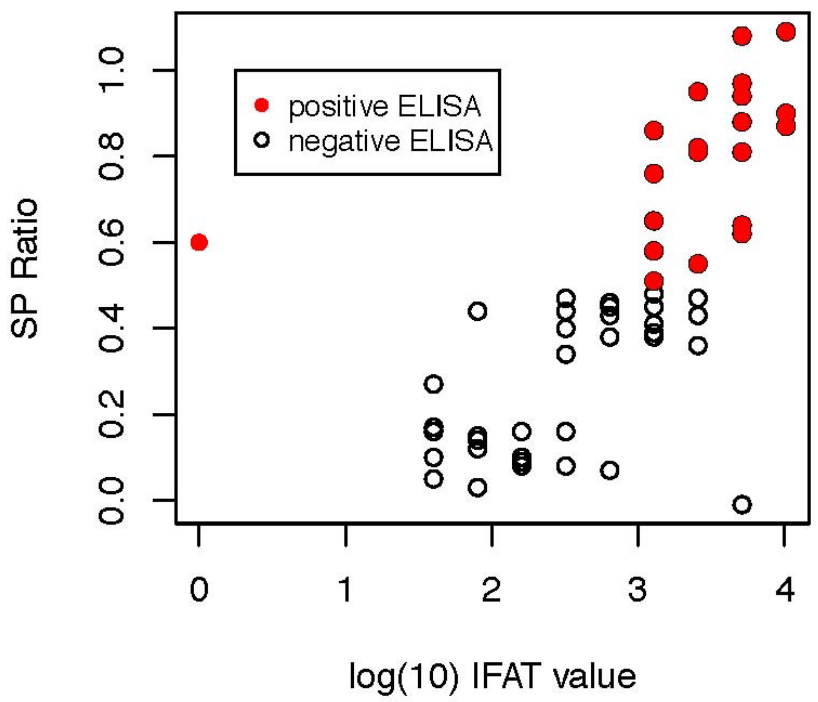

Supplementary Figure 2. Correspondence between ELISA and IFAT scores for plasma samples from 60 individual hyenas, which were tested using both methods to help corroborate our ELISA diagnostic methods. Source data are provided as a Source Data file.

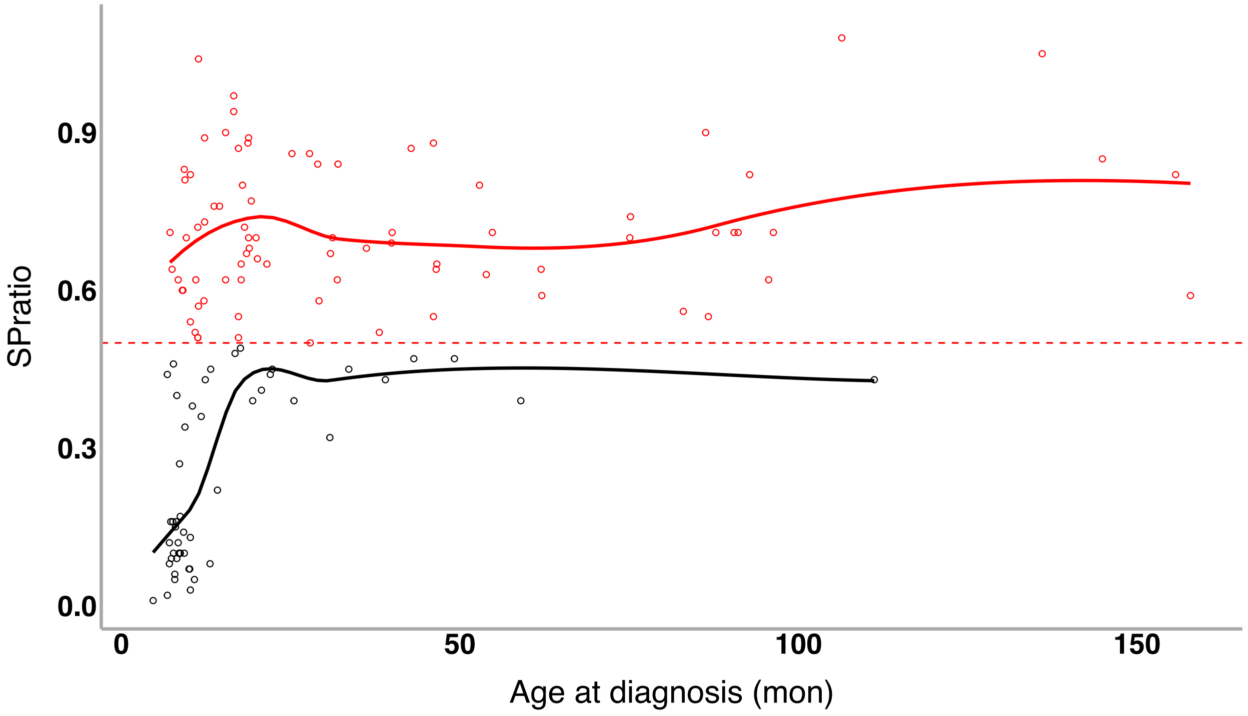

Supplementary Figure 3. SP ratio for *T. gondii* ELISA by spotted hyena age in months at the time of diagnoses. Red points and loess curve, above the horizontal red dashed line, correspond to positive infection, while the black points and black loess curve, below the horizontal red dashed line, correspond with negative diagnoses. Source data are provided as a Source Data file.

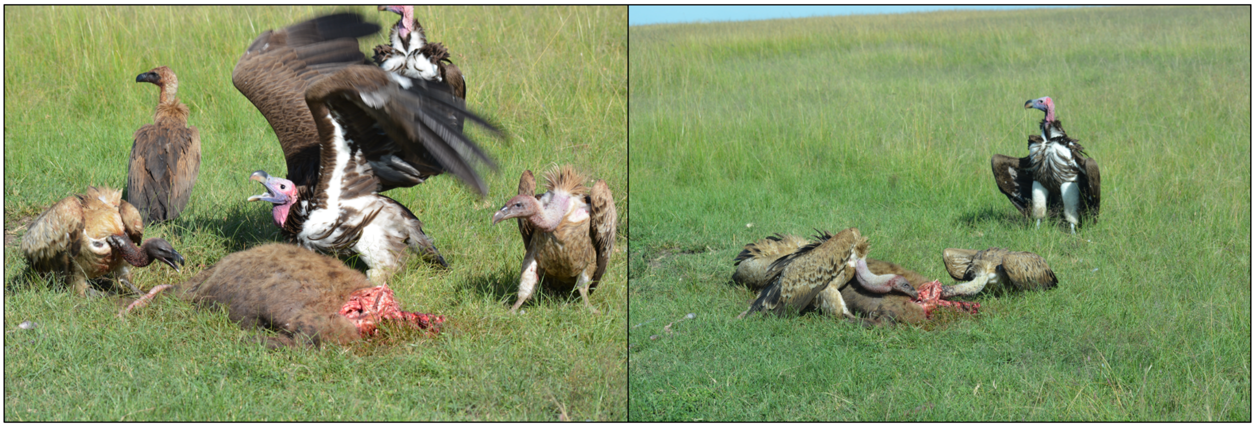

Supplementary Figure 4. Vultures eating a spotted hyena that was recently killed by a lion in the Masai Mara.

Supplementary Table 1. β (95% CI) of average minimum approach distance to lion(s) within categories of demographic and ecological characteristics among 156 hyenas from the Masai Mara, Kenya.

	β (95% CI) minimum approach distance from lions [a]
Sex	
Female	0.00 (Reference)
Male	0.47 (0.10, 0.82)*
Age at the time of hyena-lion interaction	
Cub (<12 mos)	0.0 (Reference)
Subadults (12-24 mos)	-1.92 (-2.96, -0.88)*
Adult (>24 mos)	-2.00 (-2.96, -1.05)*
Dominance Rank [b]	
Per 1 unit increment in standardized rank	-0.44 (-0.77, -0.12)*
Livestock density [c]	
High	0.00 (Reference)
Low	0.73 (0.27, 1.18)*

[a] Estimates are from a mixed linear regression model where the explanatory variables include the characteristic of interest and a random intercept for hyena ID, and the outcome is repeated assessments of a hyena's minimum approach distance (m) from lion(s) that has been square root transformed. N = 156 hyenas unless otherwise noted.

Minimum approach distances represent repeated measurements from some hyenas; total number of distance measurements = 1,190.

[b] Adult female rank or a cub's maternal rank the year during which the hyena-lion interaction was observed. On the standardized rank scale, -1 corresponds with the lowest rank and 1 with the highest rank. N = 93 hyenas.

[c] Based on illegal livestock grazing in the park during the year in which a hyena-lion interaction occurred. N = 153 hyenas.

[*] Significant at P-value < 0.05

Supplementary Table 2. The table contains a non-exhaustive list of mechanisms that we propose might generate relationships between host age, host behavior, and infection by a behavior-altering parasite (e.g., *T. gondii*).

Learning and experience modulate an infected host's behavior	Hosts that survive dangerous encounters (e.g., with predators) may subsequently upregulate inhibitory mechanisms that countervail against the behavioral influence of manipulative parasites. This possibility highlights a key limitation of lab studies involving naïve *T. gondii* (e.g., rodents) that have no interaction histories with felids.
Brain development modulates impacts of infection	Cumulative effects of infection may hinge on properties of the host brain that change throughout development into adulthood including neuroimmunology, synaptic plasticity, or neurogenesis.
Hosts of differing ages acquire infection from different sources	Among known determinants of outcomes of *T. gondii* infections are the infecting parasite life stage (oocyst vs. tachyzoite), parasite genotype, and dosage of infecting parasites (Dubey 2010). Each factor might also differ between infections of older vs. younger animals within a population due to age-dependent behaviors (e.g., diet and home range) and/or due to spatial and temporal variation in parasite population structure with respect to host age strata.
Parasites have evolved to induce different behaviors within hosts of different ages	*T. gondii* is known to infect its diverse hosts through a multiplicity of mechanisms including trophic, sexual, and vertical transmission (Dubey 2010). If the optimal transmission strategy is dependent on the host's age, the parasite might evolve divergent manipulation tactics within younger vs. older hosts.
Association between infection and behavior are diluted by other (age-dependent) variables as hosts mature	Numerous factors unrelated to *T. gondii* can regulate the behaviors of infected hosts; these potentially include co-infections with other parasites, changing social environments, differences in food requirements, etc. If these effects are stronger in older individuals, they might swamp effects of a host-manipulating parasite that are apparent in younger individuals.
