# Supplementary material for: Toxoplasma gondii infections are associated with costly boldness toward felids in a wild host
Source: Nat Commun. 2021 Jun 22;12:3842. doi: 10.1038/s41467-021-24092-x (PMC8219747; doi:10.1038/s41467-021-24092-x)
Supplement: Supplementary file 3 — Reporting Summary [file 41467_2021_24092_MOESM3_ESM.pdf]

nature research

Corresponding author(s): Zachary M. Laubach
Last updated by author(s): Apr 16, 2021

Reporting Summary

Nature Research wishes to improve the reproducibility of the work that we publish. This form provides structure for consistency and transparency in reporting. For further information on Nature Research policies, see our Editorial Policies and the Editorial Policy Checklist.

Statistics

For all statistical analyses, confirm that the following items are present in the figure legend, table legend, main text, or Methods section.

n/a	Confirmed	
☐	☒	The exact sample size (*n*) for each experimental group/condition, given as a discrete number and unit of measurement
☐	☒	A statement on whether measurements were taken from distinct samples or whether the same sample was measured repeatedly
☐	☒	The statistical test(s) used AND whether they are one- or two-sided<br>*Only common tests should be described solely by name; describe more complex techniques in the Methods section.*
☐	☒	A description of all covariates tested
☐	☒	A description of any assumptions or corrections, such as tests of normality and adjustment for multiple comparisons
☐	☒	A full description of the statistical parameters including central tendency (e.g. means) or other basic estimates (e.g. regression coefficient) AND variation (e.g. standard deviation) or associated estimates of uncertainty (e.g. confidence intervals)
☐	☒	For null hypothesis testing, the test statistic (e.g. *F*, *t*, *r*) with confidence intervals, effect sizes, degrees of freedom and *P* value noted<br>*Give P values as exact values whenever suitable.*
☒	☐	For Bayesian analysis, information on the choice of priors and Markov chain Monte Carlo settings
☒	☐	For hierarchical and complex designs, identification of the appropriate level for tests and full reporting of outcomes
☐	☒	Estimates of effect sizes (e.g. Cohen's *d*, Pearson's *r*), indicating how they were calculated

Our web collection on statistics for biologists contains articles on many of the points above.

Software and code

Policy information about availability of computer code

Data collection	Source code is provided with this paper at https://zenodo.org/badge/latestdoi/245864615 (DOI: 10.5281/zenod.4699720).
Data analysis	R version 4.0.2 (2020-06-22), Platform: x86_64-apple-darwin17.0 (64-bit), Running under: macOS Catalina 10.15.7. Packages used included: car_3.0-10 aod_1.3.1 effects_4.2-0car Data_3.0-4 emmeans_1.5.4 bbmle_1.0.23.1 lme4_1.1-26 Matrix_1.3-2 broom.mixed_0.2.6 broom_0.7.4 dotwhisker_0.5.0 gridExtra_2.3 here_1.0.1 forcats_0.5.1 stringr_1.4.0 dplyr_1.0.4 purrr_0.3.4 readr_1.4.0 tidyr_1.1.2 tibble_3.0.6 ggplot2_3.3.3 tidyverse_1.3.0

For manuscripts utilizing custom algorithms or software that are central to the research but not yet described in published literature, software must be made available to editors and reviewers. We strongly encourage code deposition in a community repository (e.g. GitHub). See the Nature Research guidelines for submitting code & software for further information.

Data

Policy information about availability of data

All manuscripts must include a data availability statement. This statement should provide the following information, where applicable:

- Accession codes, unique identifiers, or web links for publicly available datasets
- A list of figures that have associated raw data
- A description of any restrictions on data availability

Source data is provided with this paper at https://zenodo.org/badge/latestdoi/245864615 (DOI: 10.5281/zenod.4699720).

Field-specific reporting

Please select the one below that is the best fit for your research. If you are not sure, read the appropriate sections before making your selection.

☐ Life sciences ☐ Behavioural & social sciences ☒ Ecological, evolutionary & environmental sciences

For a reference copy of the document with all sections, see nature.com/documents/nr-reporting-summary-flat.pdf

Ecological, evolutionary & environmental sciences study design

All studies must disclose on these points even when the disclosure is negative.

Study description

This study uses data and samples from the Mara Hyena Project (approved by MSU IACUC), a long-term field study of individually known spotted hyenas that have been observed since May 1979. Study hyenas are monitored daily and behavioral, demographic, and ecological data are systematically collected and entered into a database. Here, we used data from four different hyena groups, called clans, as well as historic information about ecological conditions in the Masai Mara National Reserve. We maintained detailed records on the demographics of our study population, including sex, age, and the dates of key life-history milestones such as birth, weaning, dispersal and death. In the ensuing sections, we describe data collection and data processing procedures for assessment of T. gondii infection diagnosis, quantification of demographic and ecological determinants of infection status, and assessment of behavioral (boldness) and fitness (cause of mortality) characteristics hypothesized to be a consequence of positive T. gondii infection. The present analysis includes 168 hyenas, but specific subsamples vary depending on the particular hypothesis being tested.

In addition to identifying determinants and correlates of T. gondii infection, we also sought to explore the effects of infection status on hyena behavior and fitness. Over the duration of our study, we documented all observed hyena-lion encounters i.e., all instances where at least one hyena and at least one lion approached to within approximately 200m of one another. In 731 observation sessions we recorded 3,791 minimum distance estimates between individual hyenas and one or more lions along with the date, location, and identities of all hyenas present, as well as whether food (a dead prey animal or its components) or a male lion was present during the encounter, as both these factors are known to influence hyena behaviors. All boldness behaviors were extracted by four individuals blinded to infection status with 83% agreement across seven metrics recorded during hyena-lion interactions 41.

Research sample

We used data from both male and female spotted hyenas (Crocuta crocuta) from the Masai Mara, Kenya. Samples were collect from cub, subadult and adult animals (n=169) for the purpose of comparing T. gondii prevalence between males and females as well among each age group. Animals in the data set ranged in age from 2.7 months up to 236 months old.

Sampling strategy

Individually identifiable animals have been continuously observed since 1988. Behavior data are collected using focal animal surveys, critical incident, and scan sampling. Blood and other biological samples are obtained during opportunistic darting events. Based on a preliminary analyses, we determined that the 166 hyenas used in this study provided sufficient power to estimate both determinants and consequences of T. gondii infection in spotted hyenas.

Data collection

Data are collected by the project PIs (Dr. Holekamp, Dr. Smale), trained postdocs and graduate students, and trained research assistants. As part of our long-term data collection, we routinely darted study animals in order to collect biological samples and morphological measurements. Of special relevance to this study is our blood collection procedure. We immobilized hyenas using 6.5 mg/kg of tiletamine-zolazepam (Telazol ®) in a pressurized dart fired from a CO2 powered rifle. We then drew blood from the jugular vein into sodium heparin-coated vacuum tubes. After the hyena was secured in a safe place to recover from the anesthesia, we took the samples back to camp where a portion of the collected blood was spun in a centrifuge at 3000 rpm for 10 minutes to separate red and white blood cells from plasma. Plasma was aliquoted into multiple cryogenic vials. Immediately, the blood derivatives, including plasma, were flash frozen in liquid nitrogen where they remained until they were transported on dry ice to a -80°C freezer in the U.S. All samples remained frozen until time of laboratory analysis for the T. gondii assays. We measured beahvior, particularly hyena-lion interactions following a standard observation protocol. During each lion-hyena encounter, we recorded the distances between lions and individual hyenas in meters using 20-minute scan sampling of individual hyena distances from the nearest lion, as well as all-occurrence sampling of close behavioral interactions between lions and hyenas (e.g., a hyena comes within 10m of a lion). Because the body length of an adult hyena is approximately 1m, we are able to accurately estimate approach distances at this scale. Due to the inherent frenetic activity at some hyena-lion encounters, some of the minimum approach distances were recorded as ranges (e.g. 10-15m) or inequalities (e.g., <10m). For ranges, we calculated the mid-point and used this value in the analysis (e.g. if the range was 10-15m, then we used 12.5m in our calculations). If the distance range was large (>25m) and therefore highly uncertain (i.e. if the range exceeded ½ a standard deviation as estimated from the minimum approach distance data set), we removed it from the dataset. Of the 529 approach distance ranges in our data set, 225 were removed because the range exceeded 25m. We retained inequality distances by using the 'less than' distance if the recorded distance was smaller than 25m (approximately the mean [mean=45m] minus ½ a standard deviation [sd=50m] of all hyena minimum approach distances) and by including the 'greater than' distances if the recorded distance was greater than 75m (approximately the mean plus ½ a standard deviation of hyena minimum approach distances). For example, a distance recorded as <50 m would be removed from the data set as it could include a wide range of actual distances (0-50 m), while a recorded distance of <15 m was retained in the data set as 15 m. As a result of filtering inequality distances with large uncertainty, we removed 67 of 72 approach distances recorded as inequalities. Finally, we filtered the hyena approach distance to lions by removing instances when the minimum approach distance exceeded 100m, given that at this range hyenas and lions pose little threat to one another. After filtering, our final data set included 2,725 minimum approach distance estimates. It should be noted that during any particular hyena-lion interaction, we retained a single minimum approach distance for each hyena, but over their lifetime hyenas interact with lions on multiple occasions, thus the repeated minimum distance measures for individual hyenas.

Timing and spatial scale

We used behavioral data and biological samples have been collected since 1979 by the Mara Hyena Project, an ongoing field study of wild spotted hyenas (Crocuta Crocuta) in the Masai Mara National Reserve, Kenya. The specific dates of data included in this

manuscript are from 1988-09-12 through 2016-12-12. We collect data from hyenas daily barring bad weather or other limiting factors related to fieldwork. Our study is based on samples collected across this duration in order to maximize sample size variation in exposure to human disturbance.

Data exclusions

Two samples were excluded from our main analyses because of suspected assay error (e.g. one negative SP ratio and one additional IFAT vs. SP ratio discrepancy) making our final diagnostic sample size, N=166 hyenas. However, it should be noted that inclusion of these two questionable data points did not substantively change our results and had no effect on our analyses of hyena boldness or fitness as these two hyenas lacked data required for those analyses. To improve discriminatory power in our analyses that reflect these changes in livestock density and shifts in ecology, we enriched our sample selection to include hyenas from areas of low and higher livestock density. For "low livestock density," we selected animals from the eastern side of the Reserve sampled before 2000 along with animals from the western side of the Reserve (any time period). For "high livestock density," we selected animals from the eastern side of the Reserve from 2012 onward. Given the length and detail of these descriptions we point to that text instead of copying it all here.

Reproducibility

ELISA samples were performed at one time in 2018. We used a well validated commercial kit (multi-species ID Screen® Toxoplasmosis Indirect, IDVET, Montpellier) for our ELISA assays. Duplicate assays of each individual hyena yielded highly consistent SP ratios and, consequently, a repeatable T. gondii diagnosis. Similarly a single 'batch' of IFAT was conducted in 2020 for use in assay verification.

Randomization

This was an observational study that had 4 main analytical parts. H1: Greater livestock density is associated with higher risk of T. gondii infection. In this portion of the analysis, we used univariable logistic regression to investigate the relationship between livestock density (high vs. low) as the primary explanatory variable of interest, and T. gondii infection (positive vs. negative) as the outcome. In addition, we also explored associations of other key demographic characteristics as determinants of infection, namely sex, age at diagnosis and social dominance rank. Following the simple regression models that contained one single explanatory variable (unadjusted analysis), we also examined multiple-variable (mutually-adjusted) associations among the above variables. In models where dominance rank was not the primary variable of interest, we did not include rank as covariate due to missing data.
H2: Infected hyenas behave more boldly towards lions, as indicated by shorter minimum approach distances. To investigate the extent to which infection status is related to boldness behaviors, we used simple (unadjusted) and multiple-variable (adjusted) linear regression models in which T. gondii diagnosis (infected vs. uninfected) was the explanatory variable of interest, and the hyenas' square root transformed minimum approach distance (m) was the outcome. We transformed the distances to improve assumptions of normality. We stratified all models by age group such that cubs were analyzed separately from subadults and adults. We made this decision based on bivariate associations that revealed a significant age structuring of infection status (i.e., much higher prevalence of infection in subadults and adults than in cubs), as well as significant effects of age on hyena approach distances towards lions (i.e., older hyenas were much more likely to approach lions closer than younger hyenas). The cub models included individual hyenas that had both infection diagnosis and hyena-lion interaction data during their first year of life. Similarly, the subadult and adult models were restricted to include only infection diagnosis and hyena-lion interactions collected from hyenas 12 months of age and older.
When exploring associations among cubs, we first examined the unadjusted association of T. gondii diagnosis with minimum approach distance to lions. Next, we controlled for sex and age in months on the date of the interaction with lions. The age distribution of hyena cubs during observed interaction with lions were 2.7-8.5 months for uninfected cubs and 3.2 -11.8 months for infected cubs. We did not need to account for livestock density because all cubs were sampled in low livestock density areas. Additionally, for all but one cub, we only had a single minimum approach distance from lions. For the cub with multiple measures (N=3), we took the average of its minimum approach distances for use in the analysis.
When exploring associations among subadults and adults, we used a similar modeling strategy to that used for cubs, except rather than using conventional linear regression, we employed mixed linear regression models to account for the multiple assessments of minimum approach distance to lions for hyenas in these two age groups (median=5 measurements per hyena) via a random intercept for the hyena's ID. After examining unadjusted associations, we implemented a multiple variable model that adjusted for age group (subadult vs. adult) both at the time of the diagnosis and at the time of the hyena lion interaction, and sex (male vs. female). Similar to cubs, we ran sensitivity analyses that included food present during the interaction (yes vs. no), and livestock density during the year of the interaction (high vs. low). Nota bene: in the subadult and adult model age was not parametrized as a continuous measure (e.g. age in months) because for some adult female hyenas, who we began observing as adults, and for some immigrant males, whose natal clan is not known, we do not know the exact birth date of these hyenas.
In addition to the above analysis for subadult and adult hyenas, which leveraged all available hyena-lion interaction data, we also conducted sensitivity analyses on a restricted data set wherein we only considered approach distances from lions that occurred prior to the diagnostic date among hyenas who tested negative for T. gondii infection, thus ensuring these represented behaviors of uninfected hyenas. Similarly, we only considered hyena-lion interaction data that occurred after the diagnostic date for individuals who tested positive for T. gondii infection. The rationale for this approach is rooted in achieving temporal separation to avoid erroneously examining hyena-lion interactions for negative diagnosis hyenas who subsequently became infected and vice versa (nota bene: we did not do this for cubs given our small sample size in this age group and because the small age range limited the possibility that a hyena's approach from lions did not reflect its infection diagnosis). Using this restricted dataset, we modelled the associations between infection status and each hyena's closest approach distance to lions following the previously described modeling strategy.
We also modeled the hyena approach distance from lions as function of T. gondii infection among hyenas diagnosed as either positive or negative but excluding the doubtful diagnosis category. This second sensitivity analysis aimed to rule out any potential variable misclassification bias.
H3: T. gondii infection imposes fitness costs on the host, as indicated by greater odds of death by lion(s). Here, we assessed the probability that T. gondii infection in hyenas was associated with lion-induced mortality. To do this, we used logistic regression models to compare the odds of mortality due to lions vs. all other known causes of mortality for infected vs. uninfected hyenas. Following unadjusted analysis, we controlled for sex in a multiple-variable logistic regression analysis. Due to small sample sizes (i.e. cells in cross tabulations with N=0) we were not able to adjust for hyenas' ages and livestock density levels. However, we were able to use a two-by-two table and Fisher's exact test to determine whether the probability of dying by lions vs. other sources of mortality differed between infected and uninfected cubs.

Blinding

Data were collected prospectively and not for the exclusive purpose of this study. Therefore those collecting data were blind to the study design and hypotheses tested here. Diagnostic assays were performed by people who were blind to the individual hyena's demographic, ecological, and behavioral data.

Did the study involve field work? ☒ Yes ☐ No

Field work, collection and transport

Field conditions	Field conditions vary given that these data were collected over more than 30 years. In general, the Mara is a savannah ecosystem, located near the equator, and that experiences biannual wet and dry seasons.
Location	Masai Mara, Kenya, is located at approximately, latitude -1.490000 and longitude 35.143890 and an elevation of ~1600m above sea level.
Access & import/export	All sample exports are in accord with agreements between Michigan State University, the US Fish and Wildlife Serivce, Kenya Wildlife Service, and additional regional and local government authorities in Kenya.
Disturbance	Most of our data collection is non-invasive. We minimze discomfort of animals that are darted in by following our extensive darting protocol that has been approved by MSU IACUC and Kenyan Wildlife Service veterinarians.

Reporting for specific materials, systems and methods

We require information from authors about some types of materials, experimental systems and methods used in many studies. Here, indicate whether each material, system or method listed is relevant to your study. If you are not sure if a list item applies to your research, read the appropriate section before selecting a response.

Materials & experimental systems

n/a	Involved in the study	
☐	☒	Antibodies
☒	☐	Eukaryotic cell lines
☒	☐	Palaeontology and archaeology
☐	☒	Animals and other organisms
☒	☐	Human research participants
☒	☐	Clinical data
☒	☐	Dual use research of concern

Methods

n/a	Involved in the study	
☒	☐	ChIP-seq
☒	☐	Flow cytometry
☒	☐	MRI-based neuroimaging

Antibodies

Antibodies used	Using archived plasma, we diagnosed individual hyenas using the multi-species ID Screen® Toxoplasmosis Indirect kit (IDVET, Montpellier). This ELISA-based assay tests for serological (IgG) reactivity to T. gondii's P-30 antigen and has been used in many prior studies of T. gondii in diverse mammals.
Validation	A subset of 60 plasma samples, from the original 169 spotted hyenas assayed via ELISA, were also tested using IFAT diagnostics to confirm consistency between ELISA and IFAT (Figure S2; Pearson's r(58)=0.70, p<0.001).

Animals and other organisms

Policy information about studies involving animals; ARRIVE guidelines recommended for reporting animal research

Laboratory animals	No laboratory animals were used in the study
Wild animals	We used data from both male and female wild spotted hyenas (Crocuta crocuta) from the Masai Mara, Kenya. Samples were collect from cub, subadult and adult animals (n=169) for the purpose of comparing T. gondii prevalence between males and females as well among each age group. Animals in the data set ranged in age from 2.7 months up to 236 months old.<br><br>As part of our long-term data collection, we routinely darted study animals in order to collect biological samples and morphological measurements. Of special relevance to this study is our blood collection procedure. We immobilized hyenas using 6.5 mg/kg of tiletamine-zolazepam (Telazol ®) in a pressurized dart fired from a CO2 powered rifle. We then drew blood from the jugular vein into sodium heparin-coated vacuum tubes. After the hyena was secured in a safe place to recover from the anesthesia, we took the samples back to camp where a portion of the collected blood was spun in a centrifuge at 3000 rpm for 10 minutes to separate red and white blood cells from plasma. Plasma was aliquoted into multiple cryogenic vials. Immediately, the blood derivatives, including plasma, were flash frozen in liquid nitrogen where they remained until they were transported on dry ice to a -80°C freezer in the U.S. All samples remained frozen until time of laboratory analysis for the T. gondii assays.
Field-collected samples	These data were observational. As part of our long-term data collection, we routinely darted study animals in order to collect biological samples and morphological measurements. Of special relevance to this study is our blood collection procedure. We immobilized hyenas using 6.5 mg/kg of tiletamine-zolazepam (Telazol ®) in a pressurized dart fired from a CO2 powered rifle. We then drew blood from the jugular vein into sodium heparin-coated vacuum tubes. After the hyena was secured in a safe place to recover from the anesthesia, we took the samples back to camp where a portion of the collected blood was spun in a centrifuge at 3000 rpm for 10 minutes to separate red and white blood cells from plasma. Plasma was aliquoted into multiple cryogenic vials. Immediately, the blood derivatives, including plasma, were flash frozen in liquid nitrogen where they remained until they were transported on dry ice to a -80°C freezer in the U.S. All samples remained frozen until time of laboratory analysis for the T. gondii assays. Remaining samples were re-frozen at -80°C freezer in the U.S.

Ethics oversight | This study was approved by MSU IACUC

Note that full information on the approval of the study protocol must also be provided in the manuscript.
