# Supplementary material for: Toxoplasma gondii infections are associated with costly boldness toward felids in a wild host
Source: Nat Commun. 2021 Jun 22;12:3842. doi: 10.1038/s41467-021-24092-x (PMC8219747; doi:10.1038/s41467-021-24092-x)
Supplement: Supplementary file 4 — Description of Additional Supplementary Files [file 41467_2021_24092_MOESM4_ESM.pdf]

Description of Additional Supplementary Files

File Name: Supplementary Movie 1
Description: Sequence of a hyena being killed by a lion within the study's focal population (in Kenya's Masai Mara region). Although lions rarely consume hyenas after killing them, the video demonstrates a potential for *T. gondii* transmission via the ingestion of infectious blood and tissue. Footage collected by Malit Pioon April 7, 2019.
